# The Curability of Insanity

**Published:** 1887-01

**Authors:** 


### BOOK REVIEWS.

THE CURABILITY OF INSANITY: A SERIES OF STUDIES. *By* PLINY EARLE, A.M., M.D. *Octavo, pp. 227. Philadelphia:* J. B. LIPPINCOTT COMPANY. 1886. *Chicago:* A. C. MCCLURG & COMPANY.

Most of those especially engaged in the care of the insane are already familiar with the views of Dr. Earle, as they have appeared in the reports of the Northampton Lunatic Hospital for the past ten years. They will be gratified to learn that this series of studies, heretofore somewhat inaccessible, is now gathered into one volume.

Those less acquainted with asylum statistics will find in it a fair estimate of the recoveries among the insane committed to asylums. The author was led to make these studies by certain erroneous methods of tabulating recoveries in this and foreign countries. The per-centage of recoveries was formerly, and is yet in many states, based on the relation which the number of those discharged as cured bears to the whole number of admissions. Thus it came that cases of recurrent and even circular insanity furnished several "recoveries" each year. When a number of years are taken together the error is still further augmented, and we are led to regard insanity as a much more curable condition than the facts warrant.

The first paper of Dr. Earle appeared in 1876, in which he took the ground that the true measure of the curability of insanity was the proportion existing between those who recovered and the number of *individuals* treated.

The truth of this method is attested by the fact that during the past ten years it has remained substantially unchallenged, and has in many respects modified the care and provision for the insane in this and other countries.

